# Inhibition of DRP1 Impedes Zygotic Genome Activation and Preimplantation Development in Mice

**DOI:** 10.3389/fcell.2021.788512

**Published:** 2021-12-02

**Authors:** Yuanyuan Li, Ning-Hua Mei, Gui-Ping Cheng, Jing Yang, Li-Quan Zhou

**Affiliations:** ^1^ Institute of Reproductive Health, Tongji Medical College, Huazhong University of Science and Technology, Wuhan, China; ^2^ Reproductive Medical Center, Renmin Hospital, Hubei Clinic Research Center for Assisted Reproductive Technology and Embryonic Development, Wuhan University, Wuhan, China

**Keywords:** mitochondrial dysfunction, preimplantation development, zygotic genome activation, ROS, DRP1

## Abstract

Mitochondrion plays an indispensable role during preimplantation embryo development. Dynamic-related protein 1 (DRP1) is critical for mitochondrial fission and controls oocyte maturation. However, its role in preimplantation embryo development is still lacking. In this study, we demonstrate that inhibition of DRP1 activity by mitochondrial division inhibitor-1, a small molecule reported to specifically inhibit DRP1 activity, can cause severe developmental arrest of preimplantation embryos in a dose-dependent manner in mice. Meanwhile, DRP1 inhibition resulted in mitochondrial dysfunction including decreased mitochondrial activity, loss of mitochondrial membrane potential, reduced mitochondrial copy number and inadequate ATP by disrupting both expression and activity of DRP1 and mitochondrial complex assembly, leading to excessive ROS production, severe DNA damage and cell cycle arrest at 2-cell embryo stage. Furthermore, reduced transcriptional and translational activity and altered histone modifications in DRP1-inhibited embryos contributed to impeded zygotic genome activation, which prevented early embryos from efficient development beyond 2-cell embryo stage. These results show that DRP1 inhibition has potential cytotoxic effects on mammalian reproduction, and DRP1 inhibitor should be used with caution when it is applied to treat diseases. Additionally, this study improves our understanding of the crosstalk between mitochondrial metabolism and zygotic genome activation.

## Introduction

Mitochondria are dynamic organelles in the process of division and fusion and play crucial roles in many physiological functions, such as energy production, metabolites synthesis, calcium signaling, cell proliferation and death (Chan, 2012; Nunnari and Suomalainen, 2012). Mitochondrial fission is controlled by dynamin-related protein 1 (*Drp1*) (Youle and van der Bliek, 2012), which encodes the dynamin family and is recruited onto mitochondria at sites marked by endoplasmic reticulum tubules by mitochondrial dynamics factors 49 and 51, fission protein 1 or mitochondrial fission factor (Loson et al., 2013). Mitochondrial fusion is mediated by mitofusin-1 (MFN1), mitofusin-2 (MFN2) and optic atrophy (OPA1) (Youle and van der Bliek, 2012). The imbalance of mitochondrial fission and fusion contributes to mitochondrial dysfunction and structural changes, thus usually leads to harmful phenotypes *in vivo* and *in vitro*. DRP1 defects are associated with cardiomyopathy and early infant mortality in humans (Waterham et al., 2007; Ashrafian et al., 2010). Homozygous for knock-out allele of *Drp1* exhibited embryonic lethality at E11.5 with internal hemorrhage and small size (Wakabayashi et al., 2009). Brain-specific *Drp1* knockout resulted in enhanced sensorimotor gating and ectopic dendrite formation in mice (Itoh et al., 2019). Cardiac-specific *Drp1* knockout caused left ventricular dysfunction and death within 13 weeks in mice (Ikeda et al., 2015). Notably, oocyte-specific *Drp1* knockout mice were infertile by defective follicular maturation and organelle morphogenesis defects in oocytes, and this phenotype was similar like oocytes from aged mice with reduced DRP1 activity (Udagawa et al., 2014). DRP1 level was increased in brains of neurodegenerative diseases such as Huntington’s disease and Alzheimer’s disease whereas it was decreased in human lung and colon cancers (Archer, 2013; Kim et al., 2018). In addition, overexpression of *Drp1* impaired postnatal muscle growth, reduced mitochondrial DNA (mtDNA) quantity and inhibited the growth hormone pathway (Touvier et al., 2015). *Drp1* knockdown negatively influenced cell differentiation, proliferation and led to mitochondrial elongation and cell death (Uo et al., 2009; Wang et al., 2014; Parker et al., 2015).

Mitochondrial division inhibitor-1 (Mdivi-1), a selective inhibitor of DRP1 protein in mammalian cells, has pleiotropic effects in changing mitochondrial dynamics under physiological and pathological conditions (Cassidy-Stone et al., 2008). Mdivi-1 has been considered as a potential targeted therapy drug in various diseases related to mitochondrial division. On one hand, Mdivi-1 exerts cytoprotective effects in cardiac, diabetic, neural, renal tubular epithelial cells and lung macrophages by reducing mitochondrial fragmentation, oxidative stress, inflammation and apoptosis (Ong et al., 2010; Kim et al., 2017; Wang et al., 2017; Deng et al., 2020; Liu et al., 2020). On the other hand, it also has cytotoxic effects in cancer cells via decreasing cell proliferation and inducing cell apoptosis (Wang et al., 2015; Dai et al., 2020). However, treatment of normal cells with Mdivi-1 may cause impeded developmental potency. Mdivi-1 was reported to impair developmental competence and mitochondrial function of porcine embryos and fibroblast cells (Yeon et al., 2015). In addition, Mdivi-1 caused failed porcine oocyte maturation by inducing mitochondrial dysfunction, oxidative stress and apoptosis (Zhang et al., 2020). Mdivi-1 exacerbated senescence in cochlea under oxidative stress and induced hearing loss in aged mice by inhibiting mitophagy (Lin et al., 2019).

Preimplantation embryo development is an inevitable process for a fertilized egg to become a complete organism. During preimplantation embryo development, a critical event called zygotic genome activation (ZGA) occurs. ZGA event is composed of minor ZGA wave and major ZGA wave in mice. Minor ZGA occurs at the zygote stage with initiation of transcription of embryonic genes. Major ZGA takes place at the 2-cell stage with robust transcription activity. Disorders impairing ZGA may lead to 2-cell blockage in mice. ZGA activity can be modulated by transcription factors such as SOX2 (Pan and Schultz, 2011), OCT4 (Fukuda et al., 2016), NFYA (Lu et al., 2016) and DUX (Guo et al., 2019), and impacted by histone modifying enzymes which regulate active histone marks like H3K4me3, H3K27ac, H3K36me3 (Xu et al., 2019; Dahl et al., 2016) and inactive histone marks like H3K9me3 (Matoba et al., 2014), H3K27me3 (Bai et al., 2019).

Although DRP1 inhibition impairs mammalian oocyte maturation and causes meiosis arrest by mitochondrial dysfunction, little is known about the effects and mechanism of DRP1 on preimplantation embryonic development. In this study, we determined for the first time that DRP1 inhibition led to developmental arrest of preimplantation embryos in a dose-dependent manner in mice. DRP1 inhibition resulted in 2-cell arrest by ZGA inhibition and cell cycle block due to mitochondrial dysfunction.

## Materials and Methods

### Animals

Adult male and female ICR mice were purchased from SPF Biotechnology Co., Ltd (Beijing). All the mice were housed in the SPF animal facility at Huazhong University of Science and Technology.

### Collection of Mouse Zygotes for Preimplantation Development

Female ICR mice (3–4 weeks) were super-ovulated by injection with 10 IU of pregnant mare serum gonadotropin (PMSG), followed by injection of 10 IU of human chorionic gonadotropin (hCG) 48 h later. Super-ovulated female mice were mated with adult ICR male mice at a 1:1 ratio and zygotes were collected 16 h later from oviduct ampulla of female mice with vaginal plugs into M2 medium. Granulosa cells were digested by hyaluronidase and then zygotes were cultured in KSOM medium or KSOM medium containing Mdivi-1 (dissolved by DMSO for dilution to designated concentration) at 37°C in humidified atmosphere of 5% CO_2_. Melatonin (Sigma, USA), N-acetyl-cysteine (Sigma, USA) and resveratrol (Sangon Biotech, China) were used to rescue Mdivi-1-induced developmental arrest.

### Microinjection

For knockdown experiments, *Drp1* siRNA oligo (antisense sequence: 5′-AUA​UAC​GCU​AGC​UCA​AUU​GCC-3′) and control siRNA oligo (antisense sequence: 5′-ACG​UGA​CAC​GUU​CGG​AGA​ATT-3′) were synthesized (GenePharma, Shanghai, China), diluted with water to provide a working concentration of 25 μM, and approximately 5–10 pl of oligo were microinjected into the cytoplasm of early zygotes using Eppendorf Femto-Jet (Eppendorf, Germany) with Nikon Diaphot Eclipse TE300 inverted microscope (Nikon, Japan).

### Immunofluorescence and Confocal Microscopy

Embryos were fixed in 4% paraformaldehyde for 30 min at room temperature, permeabilized with PBS and 0.3% PVP containing 0.5% Triton X-100 at room temperature for 20 min, and blocked in PBS and 0.3% PVP containing 1.0% BSA at room temperature for 1 h. These embryos were incubated with primary antibody in blocking solution overnight at 4°C. After washing 3 times with PBS and 0.3% PVP, the embryos were incubated with goat anti-rabbit IgG secondary antibody at room temperature for 2 h. The embryos were washed 3 times with PBS and 0.3% PVP, stained with 10 μg/ml Hoechst 33342 containing PBS and 0.3% PVP and examined under confocal microscope (Zeiss LSM 780 META). Images were processed by ZEN software. The antibodies used are listed in Supplementary Table S1.

### Assay of Mitochondrial Membrane Potential

The 2-cell embryos were incubated with JC-1 (Beyotime, China) at 37°C in 5% CO_2_ for 30 min. The membrane potential was measured as the ratio of red fluorescence (J-aggregates) to green fluorescence (J-monomers). Fluorescence was visualized by confocal microscopy.

### Active Mitochondrial Staining

The 2-cell embryos were incubated with 500 nmol/L Mito Tracker Red CMXRos (Beyotime, China). After three washes with M2 medium, they were stained with 10 μg/ml Hoechst 33342 containing M2 medium and observed by confocal microscopy. Mito Tracker Red localization was detected by plot profile analysis with ImageJ software.

### Reactive Oxygen Species Measurement

Reactive Oxygen Species Assay Kit (S0033S, Beyotime) was applied to detect total ROS in 2-cell stage. Briefly, the 2-cell embryos cultured for 20 h were incubated in M2 medium containing 10 µM DCFH-DA at 37°C for 20 min and washed 3 times with M2 medium. Fluorescence signals were detected by fluorescent inverted microscope. ROS signals were located and quantified by ImageJ software.

## 5′-Ethynyl-2′-Deoxyuridine Assay

DNA synthesis was assessed with a BeyoClick EdU Cell Proliferation Kit with Alexa Fluor 488 (Beyotime, China). 2-cell embryos were incubated with 10 µM EdU for 2 h at 37°C and then fixed with 4% paraformaldehyde for 20 min, followed by 0.5% Triton X-100 for 20 min at room temperature. After washing with PBS for three times, click reaction solution was added according to the manufacturer’s instructions and embryos were incubated for 30 min in the dark at room temperature. Embryos were washed again and stained with Hoechst 33342 for 10 min at room temperature. The samples were imaged with confocal microscope.

### mtDNA Copy Number Measurements

mtDNA copy number refers to the number of mitochondria in the genome per cell and is a minimally proxy measure for mitochondrial function. Pool of 10–20 2-cell embryos were transferred to 20 µl lysis buffer (50 mM Tris-HCl, 200 μg/ml proteinase K, 0.5%Triton X-100) at 55°C for 2 h. Real-time PCR was performed to measure mtDNA copy number. Each experiment was repeated at least three times independently. DNA levels were normalized to *Gapdh* locus.

### ATP Measurements

ATP content was measured by the ATP determination kit (A22066, Molecular Probes) according to the manufacturer’s instructions. Briefly, 10–20 2-cell embryos were collected into 30 µl lysis buffer (20 mM Tris, 0.9% Nonidet-40 and 0.9% Tween 20). 10 µl samples were added to 96-well plates and 100 µl standard reaction solution was added into each well subsequently. The light signal was integrated by microplate reader. The light intensity was set as 1 in the control group, and the relative intensity of the treatment group was measured and compared to the control group.

### RNA Extraction and Quantitative Real-Time PCR

Total RNA was extracted from 20–30 embryos by Trizol reagent (Invitrogen, USA). RNA was precipitated by glycogen and reverse transcribed into cDNA with the Hifair 1st Strand cDNA Synthesis SuperMix for qPCR (YEASEN, China). Real-time PCR was performed in the ABI 7500 PCR system (Applied Biosystems, USA). mRNA levels were normalized to *Gapdh*. Each experiment was repeated at least three times independently. The primers used are shown in Supplementary Table S1.

### Low-Input RNA Sequencing and Data Analysis

Mouse zygotes were isolated and treated with or without Mdivi-1 for preimplantation development. A total of 10–20 2-cell embryos in each group were prepared for following RNA sequencing library preparation after culture with or without Mdivi-1 for 20 h. For library preparation, generally, samples were collected in tubes with lysis component and ribonuclease inhibitor. Then amplification was carried out by the Smart-Seq2 method. An Oligo-dT primer was introduced for first-strand cDNA synthesis, followed by PCR amplification for cDNA enrichment and purification by magbeads. The cDNA was sheared randomly by ultrasonic waves, followed by DNA fragmentation, end repair, 3’ ends A-tailing, adapter ligation, PCR amplification and library validation. Qualified libraries were loaded onto Illumina Hiseq platform for PE150 sequencing. Raw reads were processed with Cutadapt v1.16. Trimmed reads were mapped to mouse genome (GENCODE release M23) using STAR with default settings. Differential expression of genes for pairwise comparisons was assessed by DESeq2 with internal normalization of reads. Processed RNA-seq data is in Supplementary Table S2. Database for Annotation, Visualization and Integrated Discovery tools (https://david.ncifcrf.gov/) were used to analyze the enrichment of differentially expressed genes (DEGs). GO and KEGG pathway were plotted by http://www.bioinformatics.com.cn.

### Statistical Analysis

All calculations were performed by GraphPad Prism7 and all data were subjected to Student’s t test. Every experiment was repeated at least 3 times. Significance was set at *p*-value <0.05 (*p < 0.05, **p < 0.01, ***p < 0.001, ****p < 0.0001).

## Results

### mRNA and Protein Level of *Drp1* Gene During Mouse Early Embryo Development

**FIGURE 1:**
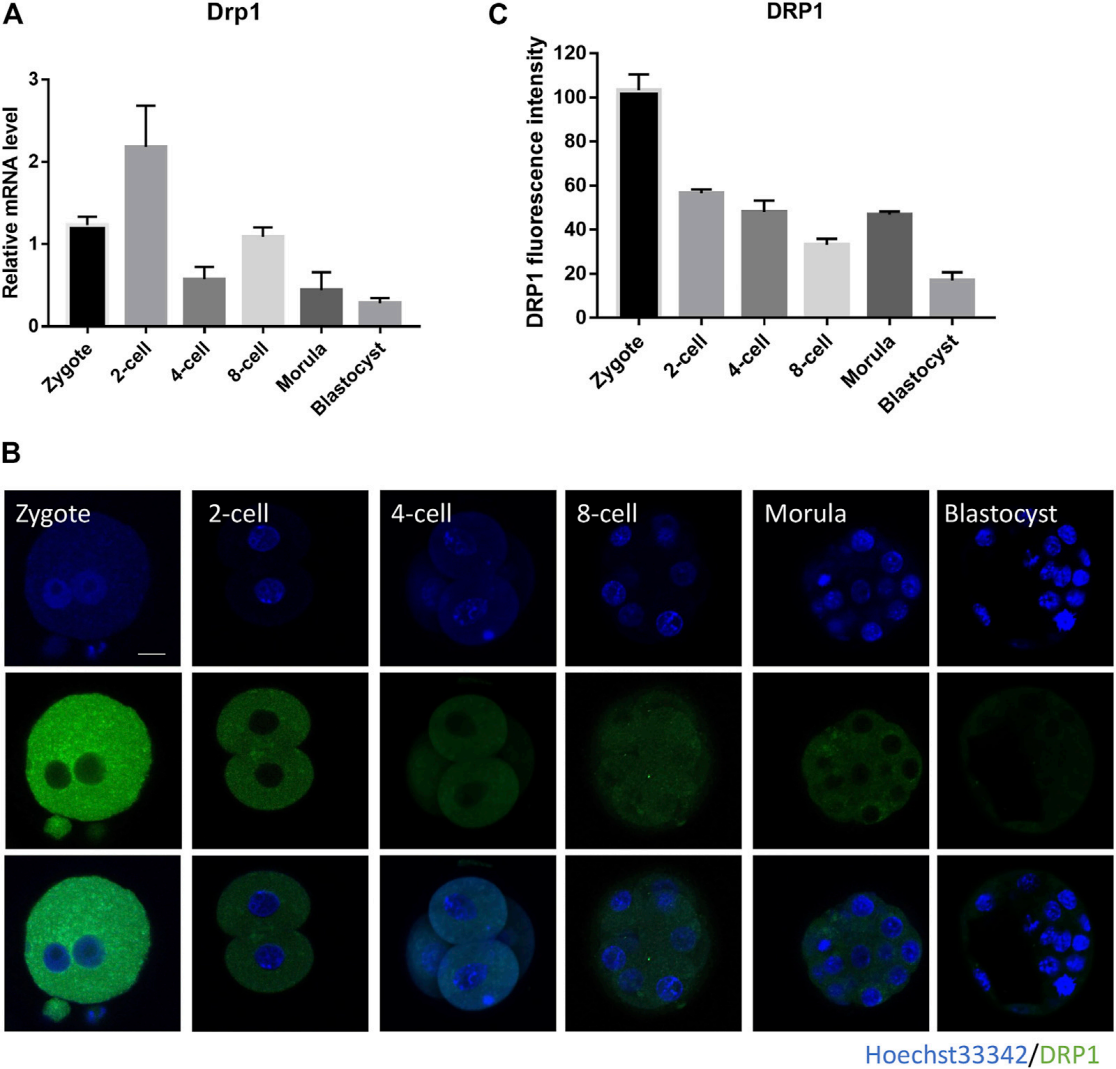
Subcellular localization and expression of DRP1 during mouse preimplantation embryonic development. **(A)** The mRNA levels of *Drp1* in mouse embryos at zygote, 2-cell, 4-cell, 8-cell, morula and blastocyst stages were examined by RT-qPCR. 20–30 embryos were collected for each group. **(B)** Mouse embryos at zygote, 2-cell, 4-cell, 8-cell, morula and blastocyst stages were immunolabeled with anti-DRP1 antibody. Green, DRP1; blue, DNA. Bar = 20 µm. **(C)** The protein levels of DRP1 in embryos at zygote, 2-cell, 4-cell, 8-cell, morula and blastocyst stages were measured by fluorescence intensity with ImageJ (*n* = 10 for each group). Data are presented as the mean ± SD from independent experiments.

mRNA and protein profiles of *Drp1* were determined by real-time quantitative PCR and immunofluorescence staining, respectively. Transcripts of *Drp1* were highly expressed in mouse early embryos, with high expression at zygote stage, became strongest at 2-cell stage and weakest at blastocyst stage (Figure 1A). Immunofluorescence staining showed that DRP1 protein is localized in the cytoplasm of preimplantation embryos (Figure 1B), and the fluorescence intensity of DRP1 is strongest in the zygote stage and became weakest in the blastocyst stage (Figures 1B,C). Inconsistent expression level of *Drp1* mRNA and protein can be explained by uncoupled transcription and translation activities during early embryonic development, and DRP1 protein level in early embryos is determined not only by mRNA level, but also maternal stored protein level, translation activity, and protein degradation activity, etc. The expression profile of DRP1 implied that DRP1 may play an important role during early embryonic development.

### Inhibition of DRP1 Led to Early Embryonic Development Arrest

**FIGURE 2:**
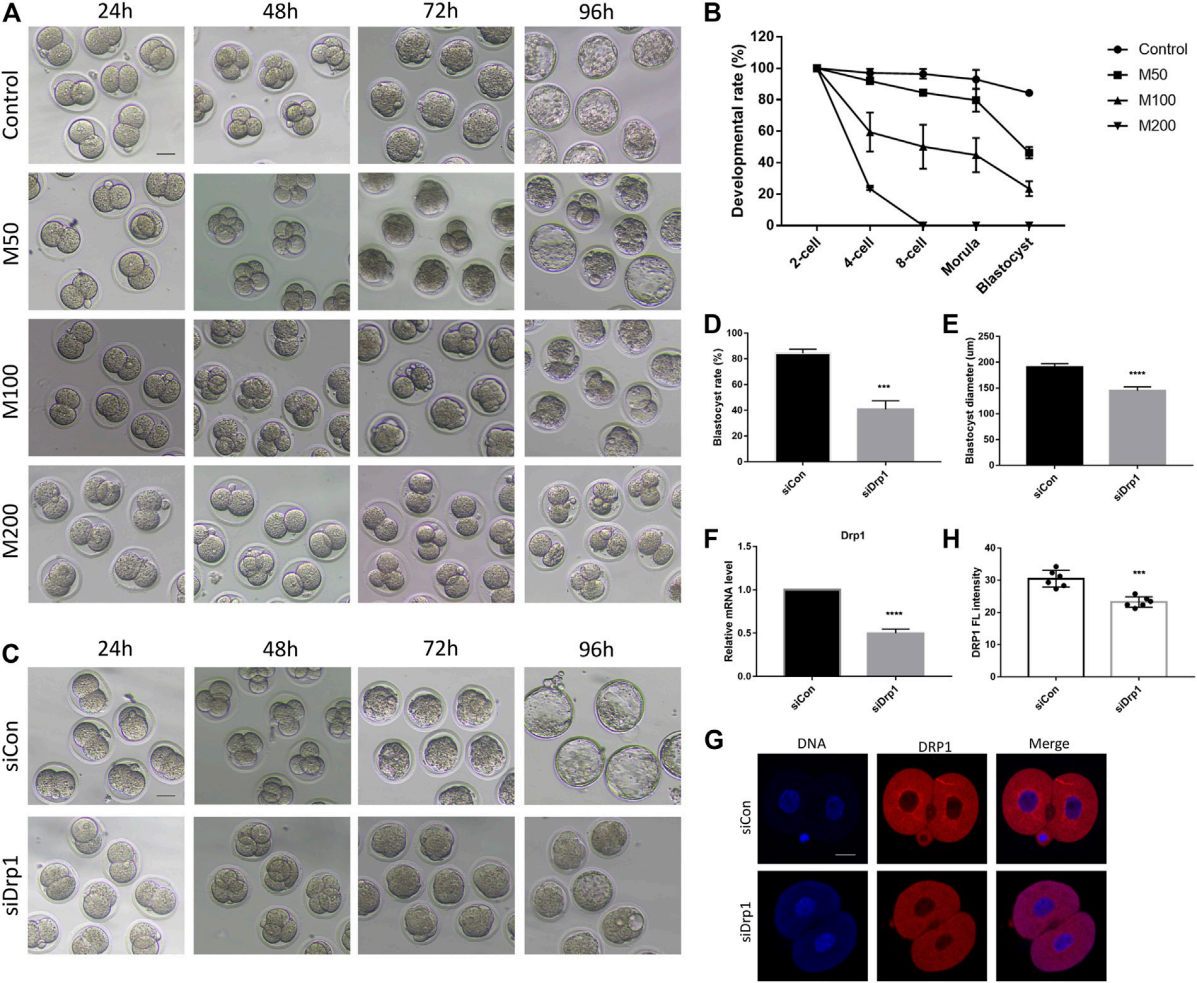
DRP1 inhibition impaired developmental competence of mouse preimplantation embryos. **(A)** DIC images of early embryonic development in control, 50,100, and 200 µM Mdivi-1 treatment groups after culturing for 24, 48, 72, and 96 h, respectively. Bar = 50 µm. **(B)** Developmental rate of different stages in early embryonic development of control and Mdivi-1 treatment groups. **(C)** DIC images of early embryonic development at 24, 48, 72, and 96 h after microinjection of control siRNA or *Drp1* siRNA into early zygotes, respectively. Bar = 50 µm. **(D)** The rate of blastocyst formation after 96 h culture of control/*Drp1* siRNA injection group. **(E)** The diameter of blastocysts after 96 h culture of control/*Drp1* siRNA injection group. **(F)** The relative mRNA levels of *Drp1* by RT-qPCR in 2-cell embryos in control/*Drp1* siRNA injection group. **(G)** Immunofluorescence staining of DRP1 in 2-cell embryos in control/*Drp1* siRNA injection group. **(H)** Fluorescence intensity quantification of DRP1 in 2-cell embryos in control/*Drp1* siRNA injection group. Bar = 20 µm ***p < 0.001; ****p < 0.0001.

To investigate the effects of DRP1 inhibition on developmental competence, we inhibited DRP1-dependent mitochondrial fission by Mdivi-1 and silenced *Drp1* by siRNA. On one hand, we collected and cultured zygotes with different concentrations of Mdivi-1 including 50, 100, and 200 µM (designated as M50, M100, M200 group, respectively). The development rates of 4-cell embryos, 8-cell embryos, morula and blastocysts were determined (Figures 2A,B). The morula and blastocyst formation rate of M50 group was significantly lower than that of the control (Figures 2A,B). The 4-cell rate was significantly decreased in M100 group while most embryos that survived until the 8-cell stage could develop further to the morula stage (Figures 2A,B). Moreover, the development rates of morula and blastocysts of M100 was significantly lower than that of M50 (Figures 2A,B). Notably, none of the embryos could develop beyond 4-cell stage and most of them were blocked in 2-cell stage in M200 group (Figures 2A,B). The results suggested Mdivi-1 treatment could disrupt the developmental competence of mouse embryos in a dose-dependent manner. On the other hand, we microinjected *Drp1* siRNA into zygotes and then found nearly all embryos can develop into morula but only a part of them developed into smaller blastocysts compared with that in the control group (Figures 2C–E). *Drp1* expression was examined by RT-qPCR (Figure 2F) and immunofluorescence (Figures 2G,H) to evaluate silencing efficiency at 24 h after microinjection. Incomplete depletion of DRP1 protein in 2-cell stage may be explained by maternally stored DRP1 protein. Therefore, we used Mdivi-1 treatment for DRP1 inhibition in further examination.

Meanwhile, we demonstrated that Mdivi-1 treatment impaired developmental competence of mouse oocytes in a dose-dependent manner. M200 showed a GVBD rate similar to that of oocyte-specific *Drp1* knockout mice (Supplementary Figure S1). Therefore, we selected 200 µM as the working concentration in subsequent studies.

### DRP1 Inhibition Resulted in Mitochondrial Dysfunction in 2-Cell Embryos

**FIGURE 3:**
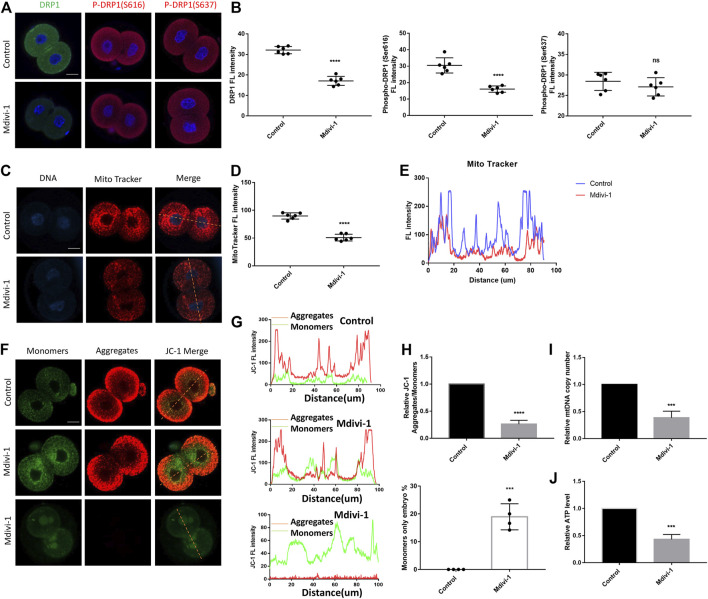
DRP1 inhibition resulted in mitochondrial dysfunction in 2-cell embryos. **(A)** Immunofluorescence staining of DRP1, P-DRP1(S616) and P-DRP1(S637) in 2-cell embryos treated with or without Mdivi-1. **(B)** The fluorescence intensity quantification of DRP1, P-DRP1(S616) and P-DRP1(S637) in 2-cell embryos treated with or without Mdivi-1. **(C)** 2-cell embryos treated with or without Mdivi-1 were labeled with Mito-Tracker Red to visualize active mitochondrial expression and localization. **(D)** The fluorescence intensity quantification of Mito-Tracker Red in 2-cell embryos treated with or without Mdivi-1. **(E)** The fluorescence intensity distribution of Mito-Tracker Red in 2-cell embryos treated with or without Mdivi-1. **(F)** Representative images of mitochondrial membrane potential assessed by JC-1 dye. **(G)** The graph showing fluorescence intensity distribution of JC-1 aggregates/monomers. **(H)** Histogram showing the JC-1 aggregates/monomers fluorescence ratio. **(I)** The relative mtDNA copy number from control and Mdivi-1 embryos (*n* = 10 for each group). **(J)** Quantitative analysis of the relative ATP content from control and Mdivi-1 embryos (*n* = 10 for each group). Data are presented as the mean ± SD from independent experiments. ns represents no statistical difference, ***p < 0.001; ****p < 0.0001. Bar = 20 µm.

Zygotes were cultured with KSOM with or without Mdivi-1 at the concentrations of 200 µM. 24 h later, 2-cell embryos were used to examine the effect of Mdivi-1 on DRP1. The results showed that Mdivi-1 significantly decreased the protein level of DRP1 (Figures 3A,B). Furthermore, Mdivi-1 suppressed phosphorylation of DRP1 at serine 616, which is required for DRP1-mediated mitochondrial fission, but not phosphorylation of DRP1 at serine 637 (Figures 3A,B). Therefore, Mdivi-1 induced DRP1 deficiency by downregulating the expression and activity of DRP1 in the study. DRP1 deficiency was reported to cause mitochondrial morphogenesis defects and dysfunction, we decided to further assess the effects of Mdivi-1 on mitochondrial function. Active mitochondria were labeled with Mito-Tracker Red CMXRos and the fluorescence intensity of active mitochondria was globally decreased in the Mdivi-1 treatment group, with the greatest decline in the perinuclear region (Figures 3C–E). Mitochondrial membrane potential was measured by JC-1 staining. The ratio of fluorescence intensity (aggregates/monomers) was significantly lower in the Mdivi-1 treatment group than that in control, especially around the nucleus (Figures 3F–H), which represented mitochondrial membrane potential depolarization. Among them, we found about 20% 2-cell embryos showed only the monomer form, which suggested early apoptosis in these 2-cell embryos. We next evaluated the mitochondria number in 2-cell embryos and found Mdivi-1 treatment reduced by more than half of the mtDNA copy number (Figure 3I). Moreover, ATP content was also decreased significantly in the Mdivi-1 treatment embryos compared to control embryos (Figure 3J). These results showed that Mdivi-1 treatment resulted in mitochondrial dysfunction.

### DRP1 Inhibition Changed the Transcriptome Profile of 2-Cell Embryos

**FIGURE 4:**
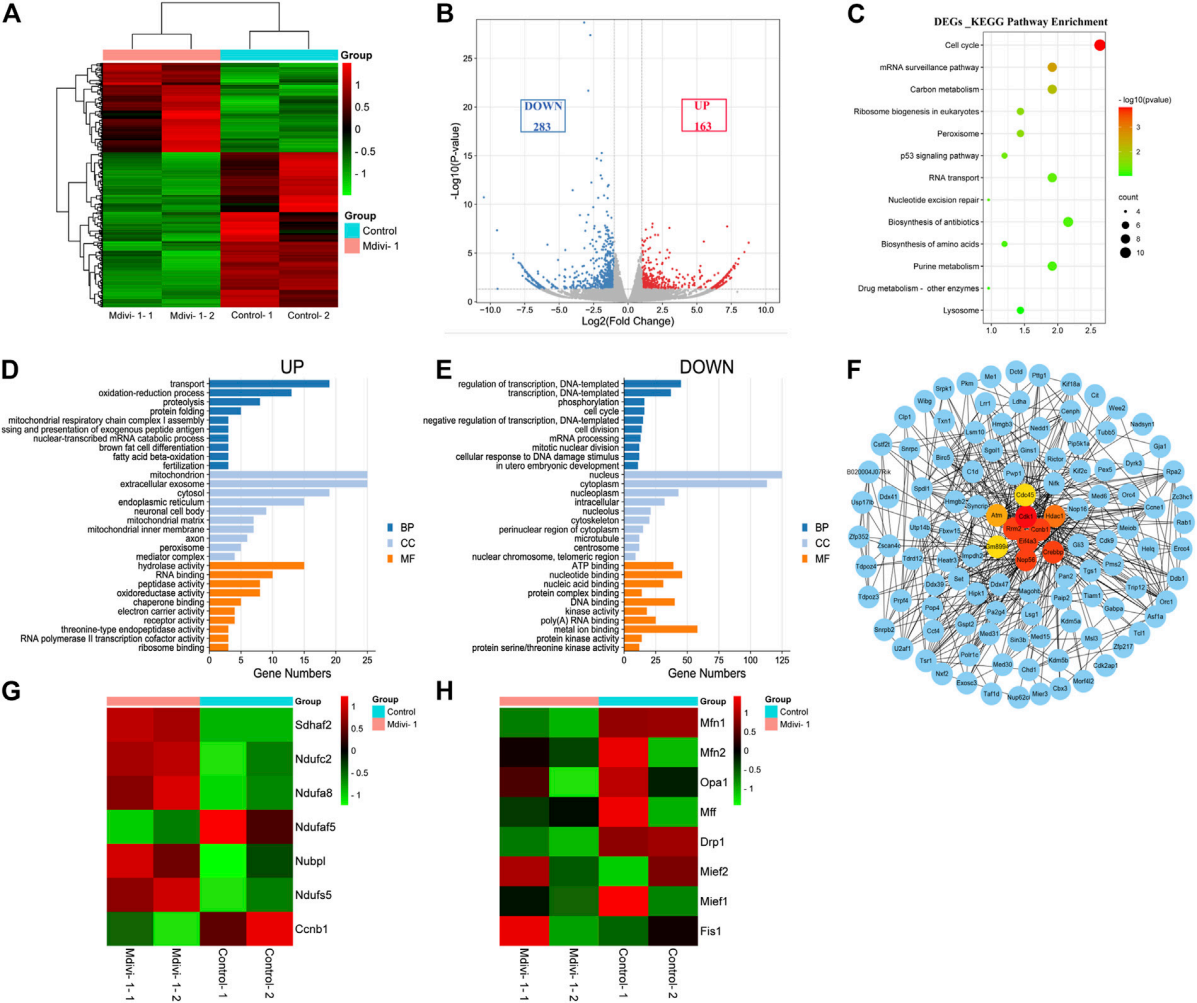
Comparison of transcriptome profiles between control and DRP1-inhibited early embryos. **(A)** Heatmap showing differential gene expression in control and Mdivi-1-treated 2-cell embryos. **(B)** Volcano plot displaying differentially expressed genes (downregulated, blue; upregulated, red) in Mdivi-1-treated embryos compared with controls. **(C)** KEGG enrichment analysis of DEGs in Mdivi-1-treated embryos compared with controls. **(D)** The top 10 highly enriched GO enrichment terms of upregulated DEGs in Mdivi-1-treated embryos compared with controls. **(E)** The top 10 highly enriched GO enrichment terms of downregulated DEGs in Mdivi-1-treated embryos compared with controls. Blue represents biological process, light blue represents cellular component and orange represents molecular function. **(F)** protein-protein interaction network of DEGs in Mdivi-1-treated embryos compared with controls. The top ten genes enriched were marked in bright colors. **(G)** Heatmap of differentially expressed genes related to mitochondrial function in 2-cell embryos treated with or without Mdivi-1. **(H)** Heatmap of genes related to mitochondrial dynamics in 2-cell embryos treated with or without Mdivi-1.

To explore the underlying mechanisms of the effects of Mdivi-1 on early embryonic program, we compared transcriptome of control 2-cell embryos with Mdivi-1 treated 2-cell embryos. Heatmap and volcano plot data reflected significant transcriptome differences of 2-cell embryos between control and Mdivi-1 groups, showing that 163 genes were upregulated and 283 genes were downregulated in Mdivi-1 treated embryos (Fold Change >1.5 and *p* < 0.05 as a cutoff) (Figures 4A,B). KEGG pathway enrichment analysis of differentially expressed genes (DEGs) involved in 13 related KEGG pathways, among which cell cycle was the most affected pathway. The result suggested that Mdivi-1 impaired cell cycle progression during early embryonic development. In addition, abnormal carbon metabolism and purine metabolism probably resulted in insufficient ATP synthesis, thus led to abnormal mRNA surveillance pathway, RNA transport, ribosome biogenesis and biosynthesis of amino acids, and then contributed to reduced activity of transcription and translation (Figure 4C). Moreover, peroxisomes are involved in excessive H_2_O_2_ production and result in oxidative stress, which could lead to DNA damage and nucleotide excision repair through P53 signaling pathway. GO enrichment analysis was also used to explore the functions of upregulated and downregulated DEGs. GO annotations were divided into three terms including biological process (BP), cellular component (CC) and molecular function (MF). To clarify the key points of analysis, we selected the top 10 enrichment items which were significantly related to BP, CC and MF. Among upregulated DEGs, oxidation-reduction process, mitochondrial respiratory chain complex I assembly and electron carrier activity were enriched and highly associated with mitochondria (Figure 4D). Among downregulated DEGs, transcription, regulation of transcription, cell cycle, cell division, mitotic nuclear division and cellular response to DNA damage stimulus were enriched and highly associated with nucleus (Figure 4E). Reduced kinase binding, poly(A) RNA binding and protein kinase binding activity are associated with regulation of transcription. Protein serine/threonine kinases has been well known as cyclin-dependent kinases to control cell cycle progression (Figure 4E). In addition, protein-protein interaction network analysis enriched top 10 genes related to cell cycle arrest due to DNA damage (Figure 4F). *Cdk1*, *Atm*, and *Ccnb1* are vital for G1/S, spindle and G2/M checkpoints, which monitor the completeness and accuracy of DNA replication. Moreover, the expression profile of genes involved in mitochondrial function was changed. Gene expression related to mitochondrial ATP synthesis coupled electron transport and mitochondrial respiratory chain complex assembly such as *Sdhaf2, Ndufc2, Ndufa8, Ndufaf5, Nubpl, Ndufs5* were dysregulated (Figure 4G). In addition, we also analyzed gene profile involved in mitochondrial dynamics and found most genes related to mitochondrial fission and fusion such as *Mff, Mfn2* and *Opa1* were not affected significantly while *Drp1* and *Mfn1* were slightly downregulated (Figure 4H). All these changes may be responsible for mitochondrial dysfunction in embryos treated with Mdivi-1.

Taken together, our RNA-seq data analysis supported that Mdivi-1 treatment led to developmental arrest at 2-cell stage by interfering with the crosstalk between mitochondrion and nucleus.

### DRP1 Inhibition Induced ROS, DNA Damage and DNA Replication Failure in 2-Cell Embryos

**FIGURE 5:**
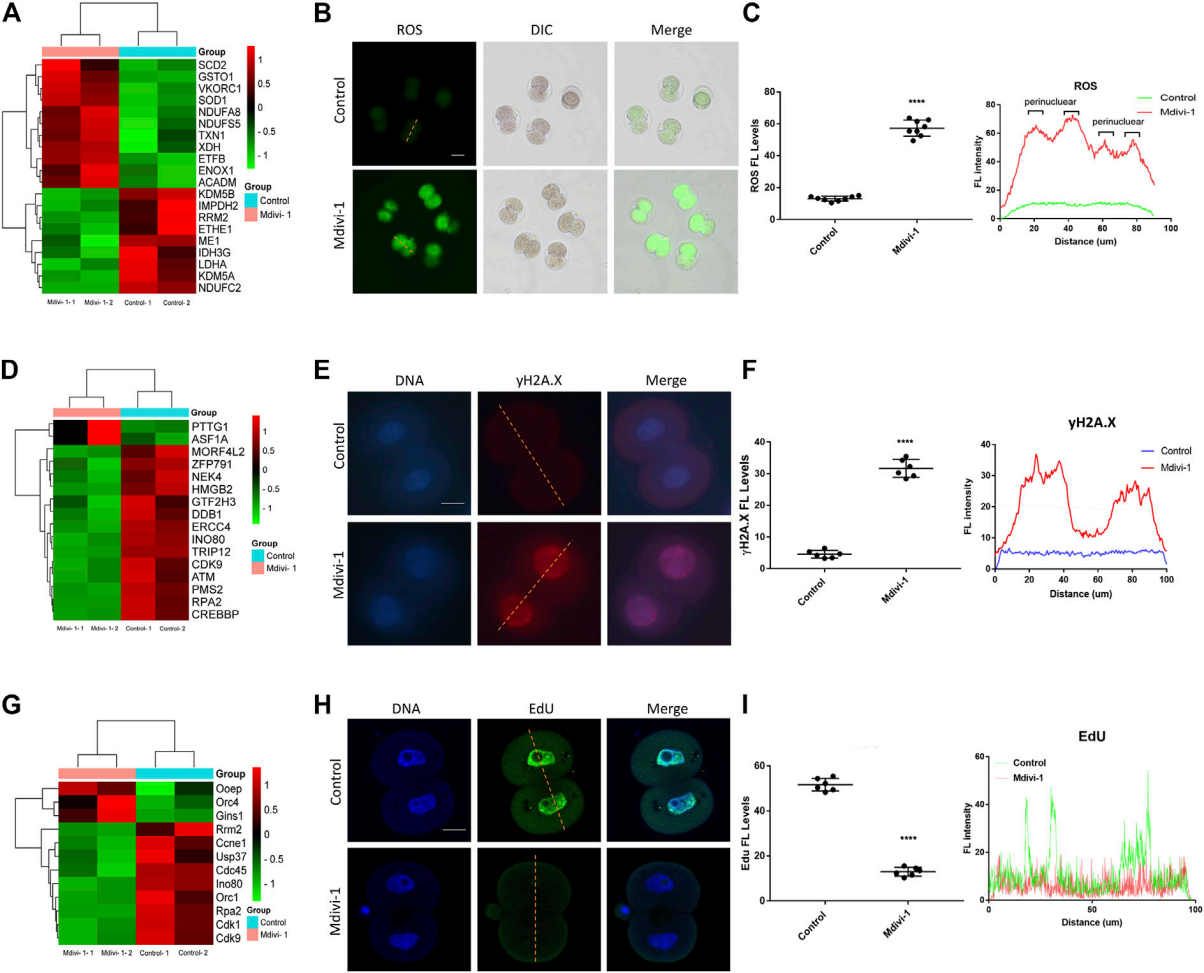
DRP1 inhibition induced ROS, DNA damage and DNA replication failure in 2-cell embryos. **(A)** The cluster analysis of differentially expressed genes related to oxidation-reduction process and oxidoreductase activity in 2-cell embryos treated with or without Mdivi-1. **(B)** 2-cell embryos treated with or without Mdivi-1 were labeled with DCFH-DA to visualize ROS level and localization. Scale bar = 50 µm. **(C)** The fluorescence intensity and distribution of ROS in 2-cell embryos treated with or without Mdivi-1. **(D)** The cluster analysis of differentially expressed genes related to DNA damage and cellular response to DNA damage stimulus in 2-cell embryos treated with or without Mdivi-1. **(E)** Immunofluorescence staining of γH2A.X in 2-cell embryos treated with or without Mdivi-1. Scale bar = 20 µm. **(F)** The fluorescence intensity and distribution of γH2A.X in 2-cell embryos treated with or without Mdivi-1. **(G)** The cluster analysis of differentially expressed genes related to DNA replication in 2-cell embryos treated with or without Mdivi-1. **(H)** 2-cell embryos treated with or without Mdivi-1 were labeled with EdU to visualize DNA synthesis. **(I)** The fluorescence intensity and distribution of EdU in 2-cell embryos treated with or without Mdivi-1. Data are presented as the mean ± SD from three independent experiments. ****p < 0.0001.

Mitochondrial dysfunction was reported to induces ROS accumulation (X. Y. Zhang et al., 2019). We next evaluated whether ROS was elevated in 2-cell embryos upon Mdivi-1 treatment. Firstly, we performed RNA sequencing of Mdivi-1 treated or untreated 2-cell embryos. RNA sequencing result revealed that 20 genes related to oxidation-reduction process and oxidoreductase activity were dysregulated in 2-cell embryos treated with Mdivi-1 (Figure 5A). As expected, further ROS examination showed that ROS level was increased globally, especially in the perinuclear regions (Figures 5B,C). Melatonin, N-acetyl-cysteine and resveratrol as antioxidant treatments were frequently used to improve oocyte and embryo quality in human and mice (Truong et al., 2016; Rodriguez-Varela and Labarta, 2020). To identify whether ROS contributed to developmental arrest, we added antioxidant melatonin, N-acetyl-cysteine or resveratrol to culture medium when embryos were treated with Mdivi-1, and found that all of them can partially rescue early embryogenesis (Supplementary Figure S2). This indicated that ROS generation was important for developmental arrest of early embryos when treated with Mdivi-1. Excessive ROS may lead to DNA damage through oxidative stress. RNA sequencing result showed that the expression levels of 16 genes related to DNA damage and cellular response to DNA damage stimulus were changed in 2-cell embryos treated with Mdivi-1 (Figure 5D). Immunofluorescence staining was performed with γH2A.X to detect DNA damage in 2-cell embryos at the same time points. The results showed that γH2A.X signals were almost undetectable in the control group while strong γH2A.X positive signals were noted in the nucleus of Mdivi-1 group (Figures 5E,F). DNA damage could destroy the integrity of the genome, damage the replication of DNA and thus prevent embryos from undergoing mitotic nuclear division. RNA sequencing results demonstrated 12 differentially expressed genes related to DNA replication in 2-cell embryos treated with Mdivi-1 when compared with control (Figure 5G). Among them, the cyclin-dependent kinase 1, CDK1, is required for G1/S and G2/M transitions of cell cycle (Malumbres and Barbacid, 2009). Fluorescence staining was performed with EdU to detect newly synthesized DNA during S phase in 2-cell embryos at 38 h post-hCG. Our results showed that EdU signals were strong in the control group but most were faint in the Mdivi-1 group, indicating that DNA replication was severely impeded upon Mdivi-1 treatment (Figures 5H,I). Collectively, 2-cell arrest phenotype was largely caused by cell cycle blockage induced by DNA damage.

### DRP1 Inhibition Reduced ZGA Activity and Disrupted the Transcriptional and Translational Activity in 2-Cell Embryos

**FIGURE 6:**
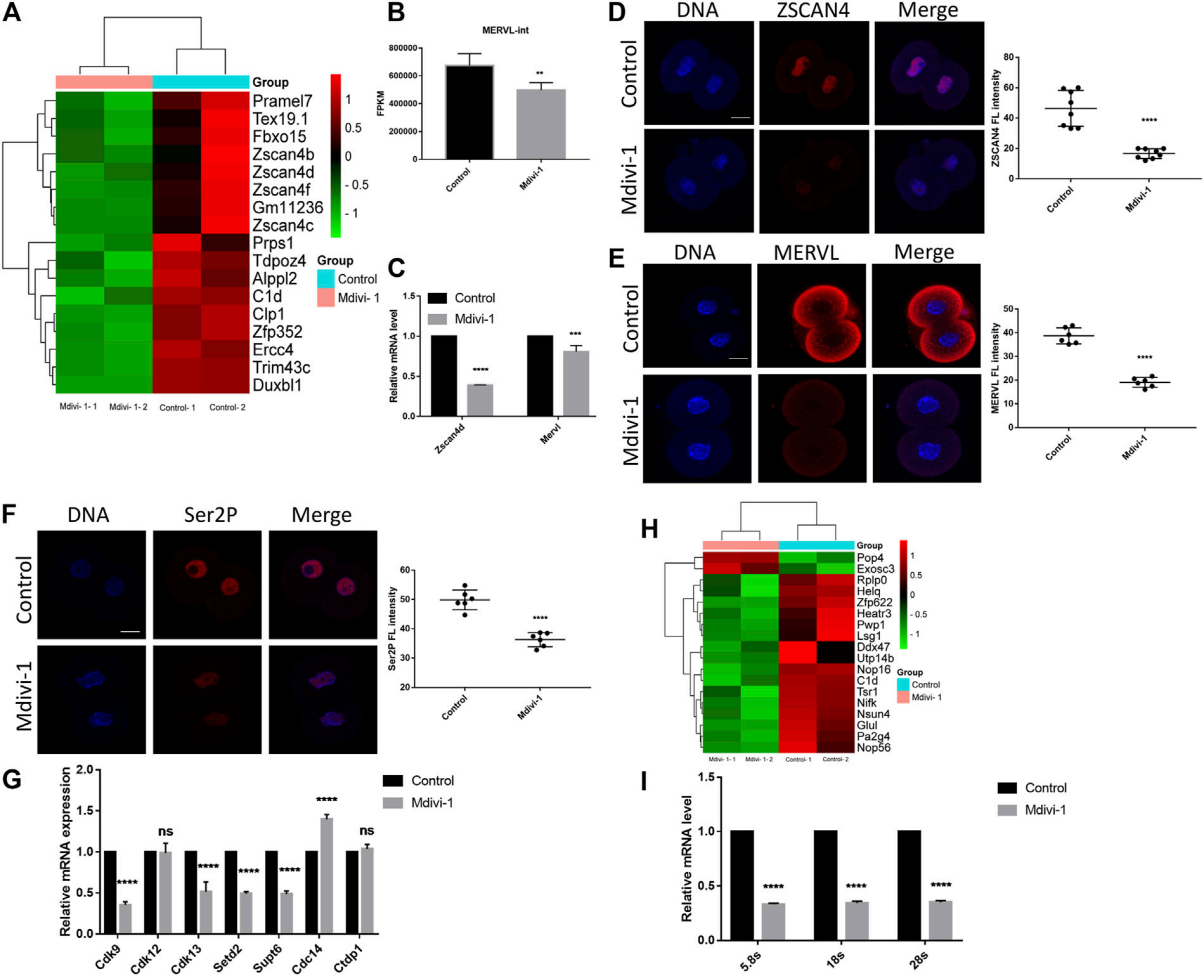
DRP1 inhibition reduced ZGA and the transcription and translation activity in 2-cell embryos. **(A)** The cluster analysis of differentially expressed genes related to ZGA in 2-cell embryos treated with or without Mdivi-1. **(B)** A histogram showing MERVL-int expression in 2-cell embryos treated with or without Mdivi-1 by RNA-seq. **(C)** The relative mRNA levels of *Zscan4d* and *Mervl* by RT-qPCR in 2-cell embryos treated with or without Mdivi-1. **(D)** Immunofluorescence staining and the fluorescence intensity of ZSCAN4 in 2-cell embryos treated with or without Mdivi-1. **(E)** Immunofluorescence staining and the fluorescence intensity of MERVL in 2-cell embryos treated with or without Mdivi-1. **(F)** Immunofluorescence staining and the fluorescence intensity of Ser2P in 2-cell embryos treated with or without Mdivi-1. **(G)** The relative mRNA levels of writers (*Cdk9*, *Cdk12* and *Cdk13*), readers (*Setd2* and *Supt6*) and erasers (*Ctdp1* and *Cdc14*) of Ser2P in 2-cell embryos treated with or without Mdivi-1 by RT-qPCR. **(H)** The cluster analysis of differentially expressed genes related to ribosome biogenesis in 2-cell embryos treated with or without Mdivi-1. **(I)** The relative mRNA levels of 5.8, 18 and 28S in 2-cell embryos treated with or without Mdivi-1 by RT-qPCR. Data are presented as the mean ± SD from three independent experiments. **p < 0.01; ***p < 0.001; ****p < 0.0001. Scale bar = 20 µm.

ZGA abnormalities could result in developmental arrest (Aoki et al., 1997). To reveal the effect of Mdivi-1 on embryonic transcription program, we examined the expression levels of ZGA markers in RNA-seq result and found that expression of typical ZGA transcripts was decreased significantly, such as *Zscan4* family, *Zfp352*, *Tdpoz4*, *Trim43c*, *Dux,* and *Mervl* (Figures 6A,B). In addition, RT-qPCR was performed to validate decreased ZGA transcripts including *Zscan4d* and *Mervl* (Figure 6C). Furthermore, immunofluorescence staining was performed for ZSCAN4 and MERVL proteins to validate impact of Mdivi-1 on ZGA. Our results indicated that protein levels of ZSCAN4 and MERVL were largely decreased in Mdivi-1 treatment group (Figures 6D,E). RNA polymerase (Pol) II-mediated transcription is indispensable for ZGA during early embryonic development (Abe et al., 2018). Phosphorylation of the Pol II C-terminal domain on serine 2 residues, Ser2P, accumulates throughout the gene body and especially toward the 3′ end and is characteristic of transcriptional elongation (Eick and Geyer, 2013). As expected, we identified reduced transcriptional activity by immunofluorescence staining with Ser2P (Figure 6F). To further understand the mechanism of Ser2P reduction, we determined the transcription levels of interacting proteins responsible for phosphorylation and dephosphorylation of Ser2 by RT-qPCR. According to the function in phosphorylating Ser2, recognizing and erasing phosphorylation of Ser2, the proteins are classified into “writers,” “readers,” and “erasers” (Eick and Geyer, 2013). We found that transcripts of both writers (*Cdk9* and *Cdk13*) and readers (*Setd2* and *Supt6*) were decreased while the transcript of eraser *Cdc14* was increased in 2-cell embryos treated with Mdivi-1 (Figure 6G), and this explained Ser2P reduction. Since we observed more reduction of protein levels than transcript levels of ZGA genes, we wondered if translation activity was also affected. Ribosome biogenesis controls the protein synthesis rate. We found 18 ribosome biogenesis-related genes were dysregulated in RNA-seq result (Figure 6H), and potential decline in translational activity due to reduced 5.8, 18 and 28S rRNA transcripts by RT-qPCR (Figure 6I). Collectively, we propose that Mdivi-1 treatment impeded embryonic program by disrupting both transcriptional and translational activities in 2-cell embryos, resulting in 2-cell developmental arrest.

### DRP1 Inhibition Altered Histone Modifications in 2-Cell Embryos

**FIGURE 7:**
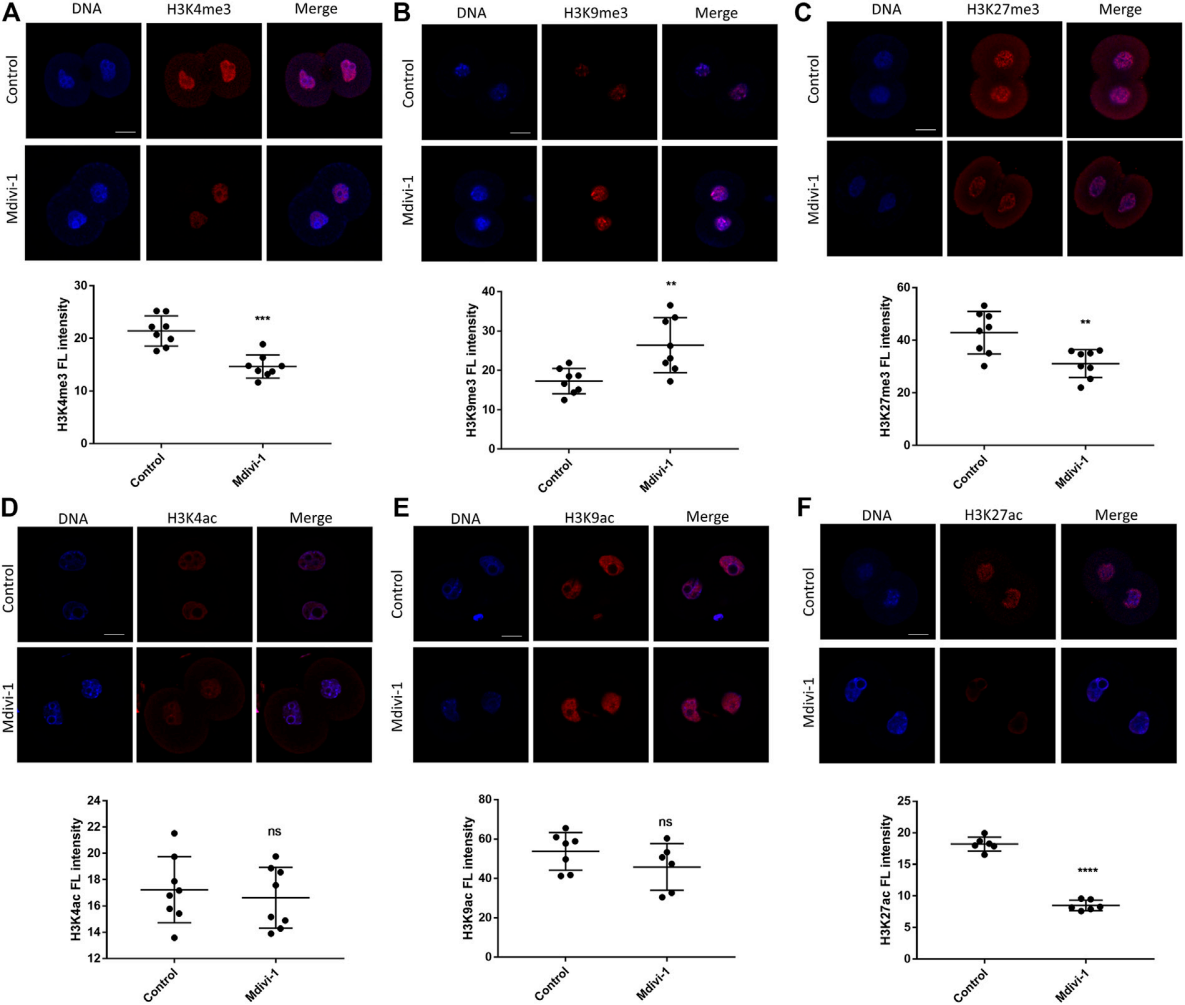
Changes of histone modifications in 2-cell embryos by DRP1 inhibition. Immunofluorescence staining and fluorescence intensity quantification of H3K4me3 **(A)**, H3K9me3 **(B)**, H3K27me3 **(C)**, H3K4ac **(D)**, H3K9ac **(E)**, and H3K27ac **(F)** in 2-cell embryos treated with or without Mdivi-1. Data are presented as the mean ± SD from three independent experiments. ns represents no statistical difference, **p < 0.01; ****p < 0.0001. Scale bar = 20 µm.

Waves of gene expression in early mouse embryos are largely orchestrated by histone modifications. Abnormal histone modifications can result in development deficiency of early embryo (Xu et al., 2021). Interestingly, mitochondrial dysfunction has been reported to exert a profound impact on histone modification dysregulation (Santos, 2021). To determine the effect of Mdivi-1 on histone modifications in 2-cell stage, we performed immunofluorescence staining of histone methylation including H3K4me3, H3K9me3 and H3K27me3 (Figures 7A–C) and histone acetylation including H3K4ac, H3K9ac and H3K27ac (Figures 7D–F). H3K4me3 and H3K27me3 were dramatically reduced and H3K9me3 was greatly increased. H3K4me3 and H3K9me3 are correlated with transcriptional initiation and heterochromatin formation, respectively, and therefore their changes were in agreement with reduced transcription activity at 2-cell stage upon Mdivi-1 treatment. Although there were no significant changes in H3K4ac and H3K9ac, H3K27ac was significantly reduced in Mdivi-1 treatment group compared with that of control, indicating specific impact of Mdivi-1 on chromatin opening status in early embryos. Taken together, our results demonstrated that Mdivi-1 altered both histone methylation and acetylation in 2-cell embryos to inhibit efficient activation of embryonic transcription program.

## Discussion

Little is known about the effects of DRP1 on mitochondrial functions during early embryonic development. Mdivi-1, a mitochondrial fission inhibitor to treat various diseases related to mitochondrial fission, could lead to mitochondrial dysfunction by DRP1 deficiency-induced abnormal mitochondrial fission and defective assembly of the electron transport chain complexes. Mdivi-1 has been reported to impair mitochondrial function and developmental competence of porcine embryos (Yeon et al., 2015). In current study, we demonstrated that DRP1 plays crucial roles in controlling the quantity and quality of mitochondria and early embryonic development in mice through directly inhibiting DRP1 GTPase activity and mitochondrial functions by Mdivi-1 treatment. As a result, Mdivi-1 supplement of embryo culturing *in vitro* resulted in blockage at 2-cell, 4-cell and morula stages.

DRP1 is a member of the dynamin superfamily of GTPases. It is critical for mitochondrial and peroxisomal division in mammals (Koch et al., 2003; Taguchi et al., 2007). Mitochondrial fission, which is required for normal cell growth and development, is regulated by the phosphorylation of DRP1 at Ser616 by Cdk1/cyclin B and at Ser637 by protein kinase A (Knott et al., 2008). Phosphorylation of DRP1 at Ser616 stimulates mitochondrial fission during mitosis while phosphorylation of DRP1 at Ser637 inhibits mitochondrial fission (Knott et al., 2008). The balance between mitochondrial fission and fusion is crucial for proper mitochondrial function. DRP1 was strongly expressed in the cytoplasm of zygote and 2-cell stages during mouse embryonic development, which suggested that DRP1 plays an important role in ZGA. In accordance with previous report (Aishwarya et al., 2020), Mdivi-1 treatment reduced the expression of DRP1 and phospho-DRP1(Ser616) in 2-cell embryos, induced mitochondrial elongation and swelling, thus impaired mitochondrial morphology and function. Active mitochondria, mitochondrial membrane potential and mtDNA copy number were decreased significantly due to impaired DRP1 self-assembly in Mdivi-1 treatment group. We noted that the decreased mRNA level of *Cdk1*, responsible for the phosphorylation of DRP1 at Ser616, may be the reason for mitochondrial dynamics imbalance and dysfunction in Mdivi-1 treated embryos. The effect and mechanism of Mdivi-1 on the expression and phosphorylation of DRP1 are complicated and need further investigation.

Mdivi-1 has been reported to inhibit respiration at mitochondrial complex I and modify electron transfer ROS production of mammalian cells in a DRP1-independent fashion (Bordt et al., 2017). Excessive ROS production is closely related to the imbalance of oxidation and reduction, DNA damage as well as developmental arrest (Kang et al., 2017). By GO enrichment analysis of RNA sequencing result we found Mdivi-1 treatment disrupted mitochondrial respiratory chain complex I assembly, which is responsible for electron transport ROS production and ATP production across the mitochondrial inner membrane (Mimaki et al., 2012). Complex I dysfunction is the most common oxidative phosphorylation (OXPHOS) disorder in mammals. Here Mdivi-1-induced ROS production and oxidative stress-induced DNA damage should be the main cause of cell cycle arrest in 2-cell embryos. Disrupted mitochondrial REDOX processes including fatty acid β oxidation (FAO) and oxidative phosphorylation (OXPHOS) lead to ATP production defects. Insufficient ATP synthesis could result in decreased activity of ATP-dependent physiological activities, such as DNA replication, mRNA transcription and protein translation, which may be responsible for ZGA defects in arrested 2-cell embryos.

Mitochondrial metabolism exerted a profound impact on histone modifications, which are required for fine-tuning of gene expression (Martinez-Pastor et al., 2013). Mitochondrial tricarboxylic acid (TCA) cycle enzymes are essential for epigenetic remodeling during ZGA (Nagaraj et al., 2017). The imbalance between active H3K4me3 and repressive H3K27me3 dysregulated chromatin state during ZGA (Liu et al., 2016). In this study, Mdivi-1 treatment interfered with lactate dehydrogenase A and isocitrate dehydrogenase 3, which may regulate ZGA. Mdivi-1 also resulted in decreased levels of both H3K4me3 and H3K27me3, while repressive histone marker H3K9me3 was increased significantly. Decreased active H3K27ac in the Mdivi-1 treated 2-cell embryos may be due to a combined effect of decreases in the histone acetylation “reader” BRD4, DRP1 S616 phosphorylation and acetyl-CoA (Chan et al., 2019; Wang et al., 2020; Zhou et al., 2021). In addition, Mdivi-1 treatment decreased *Hdac1* transcript and disrupted histone phosphorylation by downregulating the activity of kinases such as *Atm* and *Cdk9*. Moreover, Mdivi-1 treatment impaired poly(A) RNA binding and accelerated ubiquitin-dependent proteolysis, probably leading to reduced protein stability of ZGA markers such as ZSCAN4 and MERVL. These results suggested Mdivi-1-induced mitochondrial dysfunction is the major reason causing ZGA failure through histone modifications.

In summary, DRP1 inhibition by Mdivi-1 caused developmental arrest of preimplantation embryos in a dose-dependent manner in mice. DRP1 inhibition resulted in mitochondrial dysfunction by disrupting DRP1, and led to excessive ROS production, DNA damage and cell cycle arrest at 2-cell embryo stage. Reduced transcriptional and translational activity together with dysregulated histone modifications impaired ZGA activity and developmental potency. Our results demonstrate that DRP1 inhibition has cytotoxic effects on early embryonic development and provide new clues for mitochondria-related reproductive diseases.

## Data Availability

The datasets presented in this study can be found in online repositories. The names of the repository/repositories and accession number(s) can be found below: BioProject: PRJNA760437.

## Abbreviations

DRP1, dynamic-related protein 1; Mdivi-1, mitochondrial division inhibitor-1; ROS, reactive oxygen species; ZGA, zygotic genome activation.
