# Supplementary material for: Inhibition of DRP1 Impedes Zygotic Genome Activation and Preimplantation Development in Mice
Source: Front Cell Dev Biol. 2021 Dec 2;9:788512. doi: 10.3389/fcell.2021.788512 (PMC8675387; doi:10.3389/fcell.2021.788512)
Supplement: Supplementary file 3 [file DataSheet1.DOCX]

Supplementary Figures


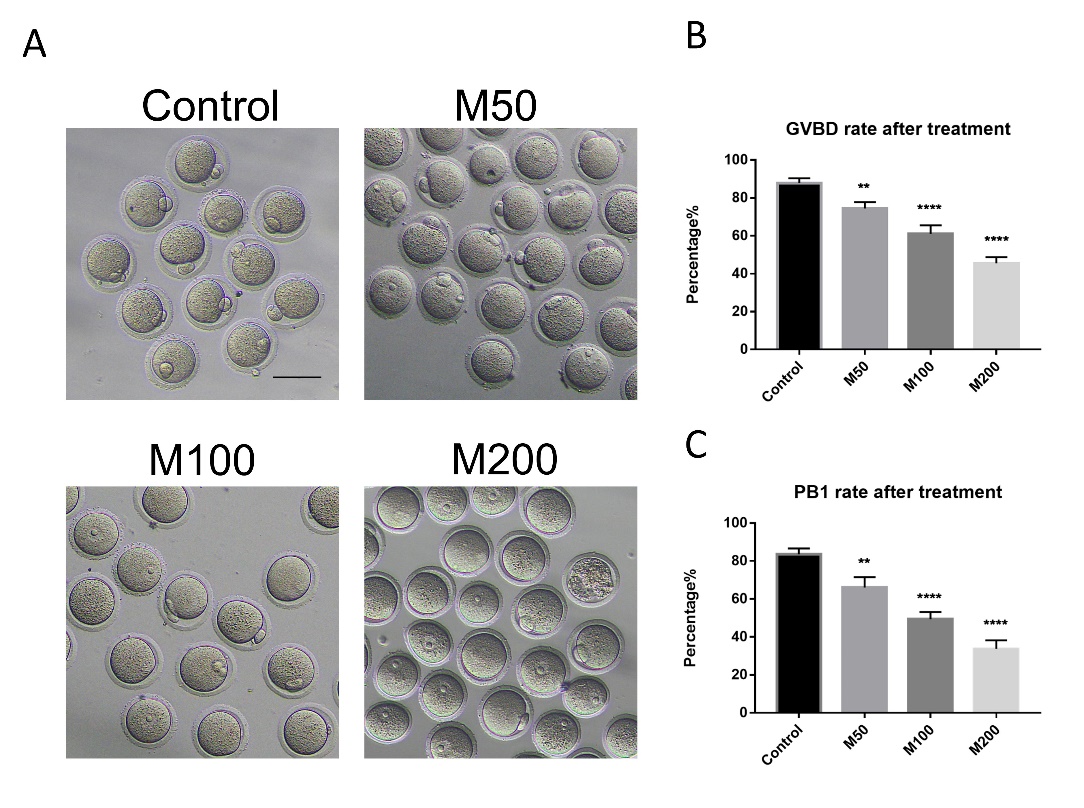


Supplementary Fig. 1. DRP1 inhibition by Mdivi-1 impaired the developmental competence of mouse oocytes. (A) DIC images of oocyte development in control, 50,100, and 200 µM (M50, M100, M200) Mdivi-1 treatment groups after culturing for 16 h, respectively. Scale bar = 100 µm. (B) GVBD rate after 16 h culture of oocyte in control and Mdivi-1 treatment groups. (C) The polar body extrusion rate after 16 h culture of control and Mdivi-1 treatment groups. N = 18–30 oocytes analyzed per group in each experiment replicated for three times. **P < 0.01; **** P < 0.0001.


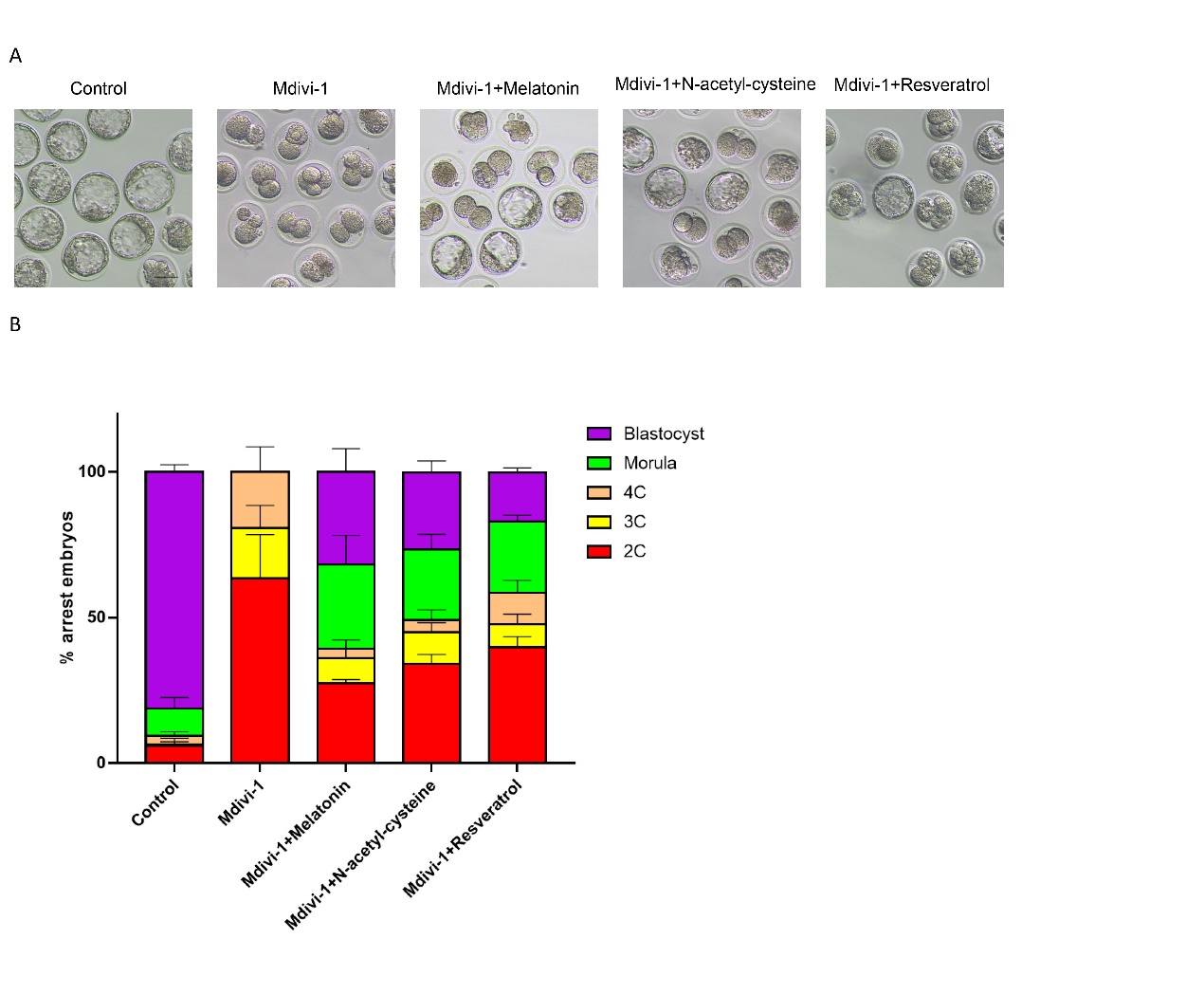


Supplementary Fig. 2. The antioxidants partially reversed Mdivi-1-induced developmental arrest of mouse embryos. (A) Representative images of preimplantation embryo development in control, Mdivi-1 (200µM) treated, melatonin (10^-9^ M), N-acetyl-cysteine (1mM) and resveratrol (0.5µM) supplemented groups at 96h after culture. Scale Bar = 50um. (B) Bar graph showing the percentage of embryos at different developmental stages in control, Mdivi-1 treatment, melatonin, N-acetyl-cysteine and resveratrol supplemented groups at 96 h after culture. N = 20–30 embryos analyzed per group in each experiment replicated for three times. Error bars indicate the mean ±SD.
